# Tadalafil 5 mg once daily for the treatment of erectile dysfunction during a 6-month observational study (EDATE): impact of patient characteristics and comorbidities

**DOI:** 10.1186/s12894-015-0107-5

**Published:** 2015-11-12

**Authors:** Dimitrios Hatzichristou, Gianluca d’Anzeo, Hartmut Porst, Jacques Buvat, Carsten Henneges, Andrea Rossi, Karim Hamidi, Hartwig Büttner

**Affiliations:** Centre for Sexual and Reproductive Health and 1st Department of Urology, Aristotle University of Thessaloniki, Thessaloniki, Greece; Medical Advisor Urology, Eli Lilly Italy S.p.A., Via A. Gramsci 731/733, 50019 Sesto Fiorentino, FI Italy; Private Practice of Urology and Andrology, Hamburg, Germany; Centre d’Etude et de Traitement de la Pathologie de l’Appa reil Reproducteur et de la Psychosomatique (CETPARP), Lille, France; Lilly Deutschland GmbH, Bad Homburg, Germany; Eli Lilly and Company, Neuilly sur Seine, France

**Keywords:** Tadalafil, Once daily use, Erectile dysfunction, Comorbidities, Observational study, EDATE

## Abstract

**Background:**

To explore the impact of patient-characteristics and relevant comorbidities on treatment continuation rates, effectiveness, and satisfaction in patients with erectile dysfunction (ED) who started or switched to tadalafil 5 mg once daily (TAD-OaD) at baseline.

**Methods:**

In the EDATE observational study, phosphodiesterase-type-5 (PDE5)-inhibitor pretreated or naïve ED patients who started or switched to TAD-OaD were prospectively followed for 6 months. Time to discontinuation of TAD-OaD was estimated using the Kaplan-Meier product-limit method at Months 2, 4, and 6 in subgroups stratified by age (18 − 65 years and >65 years), PDE5-inhibitor pretreatment, ED-severity (mild, moderate, severe), and presence or absence of relevant comorbidities (BPH, diabetes, CVD, hypertension, dyslipidemia). LSmean change from baseline in International Index of Erectile Function (IIEF) and Erectile Dysfunction Inventory of Treatment Satisfaction (EDITS) scores and associated 95 % CIs were assessed using a mixed-model for repeated measures. Visit, ED etiology, and subgroups were included as fixed-effects.

**Results:**

Overall, 778 patients received prescriptions for initiating or switching to TAD-OaD at baseline. At Month 2, >90 % of patients remained on TAD-OaD, except those aged >65 years (86.7 %) and patients with severe ED (89.0 %). More than 80 % of patients in all subgroups, except those aged >65 years (75.0 %), continued TAD-OaD at Month 6. There was a significant LSmean negative effect on IIEF- EF domain-score improvement for BPH (LSmean effect [95 % CI]: −2.77 [−4.98, −0.55], *p* = 0.014), previous PDE5-inhibitor treatment (−2.13 [−3.33,-0.94], *p* < 0.001), and mild vs moderate ED (-2.00 [−3.54,-0.46], *p* = 0.011); the latter possibly linked with a bigger treatment-effect in those with more severe ED at baseline. The LSmean effect on change in IIEF-EF was significantly positive for diabetes (2.28 [0.64,3.92], *p* = 0.007), most likely because those with diabetes had more severe ED at baseline. For all other parameters, no statistically significant LSmean effects in IIEF-EF changes were observed. No comorbidity or baseline-characteristic except age (18 − 65 years vs >65 years: 11.25 [2.96,19.54], *p* = 0.008) affected changes in EDITS.

**Conclusions:**

Under routine clinical conditions, treatment continuation rate or satisfaction does not seem to be significantly affected by the presence of comorbidities in men who choose ED-treatment with TAD-OaD. The magnitude of treatment effectiveness was affected by certain baseline characteristics and comorbid conditions.

**Trial Registration:**

The study (H6D-EW-LVIU) is registered in the German VfA Registry of Non-Interventional Studies (Verband Forschender Arzneimittelhersteller) since 06 December 2011; available at: http://www.vfa.de/de/arzneimittel-forschung/datenbanken-zu-arzneimitteln/nisdb/nis-details/_741.

**Electronic supplementary material:**

The online version of this article (doi:10.1186/s12894-015-0107-5) contains supplementary material, which is available to authorized users.

## Background

Men with erectile dysfunction (ED) have several comorbid conditions such as cardiovascular disease (CVD) and associated risk factors including diabetes, hypertension, dyslipidemia, smoking, obesity, sedentary lifestyle, and others such as depression, premature ejaculation, lower urinary tract symptoms (LUTS) secondary to benign prostatic hyperplasia (BPH), and overactive bladder [1–4]. In the Men’s Attitudes to Life Events and Sexuality (MALES) study, 64 % of the men reported having at least 1 comorbid condition including hypertension (36 %), dyslipidemia (29 %), depression (25 %), CVD (17 %), and diabetes (14 %) [1].

Given these prevalence rates of comorbidities in men with ED, it is important to choose an appropriate treatment that maintains its efficacy and tolerability in the presence of the comorbidities [5]. Oral phosphodiesterase type 5 (PDE5) inhibitors including avanafil, sildenafil, tadalafil, and vardenafil are proven to be safe and effective for managing ED [6–14]. Furthermore, these drugs have demonstrated efficacy in men with ED who have comorbid cardiovascular risk factors including hypertension, dyslipidemia, and/or diabetes [5], and tadalafil once daily (OaD) has shown efficacy in the treatment of BPH/LUTS [15–17].

It has been suggested that appropriate treatment of concomitant ED may help in improving the adherence and management of associated comorbidities, which may eventually decrease healthcare costs and improve the overall health of the affected men [18]. However, some pharmacological drugs used for treating these comorbid conditions have sexual side effects. For example, antihypertensive drugs such as thiazide diuretics increase the risk of ED; 5α-reductase inhibitors, used for treating LUTS linked to symptomatic BPH, are associated with sexual disorders; and α-blockers tamsusolin and silodosin, used for treating BPH, are frequently responsible for ejaculatory disorders like anejaculation and reduced ejaculate volume [19]. This further underscores the significance of choosing an efficacious medication for treating ED in these subpopulations, if required.

The EDATE study was the first multinational, observational study in patients with ED, with or without previous exposure to PDE5 inhibitors, that documented the effectiveness and tolerability of tadalafil 5 mg OaD under routine conditions. The study highlighted that more than 85 % of men who choose to be treated with tadalafil OaD maintained the treatment in the next 6 months [20]. In this article, we present results of secondary analyses for the impact of patient characteristics and relevant comorbidities on treatment continuation rates as well as treatment effectiveness and satisfaction in patients with ED who had started with or switched to tadalafil OaD and were followed for a period of 6 months.

## Methods

### Patients and study design

EDATE was a prospective, longitudinal, observational study conducted in 59 centres across Germany, France, Italy, and Greece, enrolling patients from November 2011 through June 2012 [20]. Adult male patients who met the investigator’s criteria for ED and presented within the normal course of care were eligible to participate if they had decided, in consultation with their physician, to either initiate PDE5 inhibitor treatment for the first time (treatment-naïve) or switch from any previous PDE5 inhibitor taken on-demand. Patients with previous experience with tadalafil OaD were excluded. The study was approved by the Comité de protection des personnes “Nord-Ouest IV”, centre hospitalier universitaire, faculté de médecine, pôle recherche, 59045 Lille Cedex, and additional ethical review boards as per individual study site and country requirements (full list provided as Additional file 1). Patients provided written informed consent for data collection, storage, and release of anonymised data.

Assessment and treatment of patients were performed as per routine investigator practice for managing ED. In this article, data from patients who initiated or switched to treatment with tadalafil OaD at baseline (Visit 1) and were followed up longitudinally for up to 6 months (tadalafil OaD cohort) are reported. Post-baseline data were collected at routine visits (Visit 2, Visit 3) within 1 to 3 months and 4 to 6 months after initiation of therapy. Patients who switched or discontinued tadalafil OaD treatment during the observation were followed until the end of the 6-month observation period. A follow-up phone call was made to patients who did not visit within 4 to 6 months after baseline.

### Outcome measures

The primary results on the time to treatment discontinuation of tadalafil OaD in men who had initiated or switched to tadalafil OaD at baseline have already been published [20]. The current article includes secondary outcome results on the impact of patients characteristics and comorbidities on time to treatment discontinuation, and on mean changes from baseline to end of observation in the International Index of Erectile Function (IIEF) domain scores [21], and Erectile Dysfunction Inventory for Treatment Satisfaction (EDITS) total score [22].

### Statistical analysis

#### Sample size

A sample size of 250 patients initiating or switching to tadalafil OaD at baseline was planned, based on the primary outcome (continuation rate on tadalafil OaD, estimated to be 50 − 80 % after 6 months of treatment) [20]. Consecutive enrollment was planned to be stopped as soon as either 3,000 patients were enrolled overall, or 600 patients were enrolled in the tadalafil OaD cohort.

#### Primary analysis

All patients prescribed tadalafil OaD treatment at baseline were included in the analysis. The distribution of time to discontinuation of tadalafil OaD was estimated using the Kaplan-Meier product-limit method. The Kaplan-Meier proportions and associated 95 % confidence intervals (CIs) of patients on tadalafil OaD at Months 2, 4, and 6 were reported for the tadalafil OaD cohort (primary analysis) as well as subgroups stratified by age (18–65 years and >65 years), pretreatment with PDE5 inhibitors, ED severity (mild, moderate, severe), and presence or absence of BPH, diabetes, CVD, hypertension, and dyslipidemia (prespecified subgroup analyses, reported in this manuscript).

An additional, prespecified exploratory analysis was performed to investigate the association between time to discontinuation of tadalafil OaD and selected baseline factors using a Cox proportional hazards model; hazard ratios (HRs) and the corresponding 95 %CIs were reported. The final model included factors associated with treatment discontinuation identified by backward selection (removing those with *p* > 0.1). These included presence of relevant comorbidities, type of physician who initially diagnosed the ED, country, duration of living arrangement, age, ED etiology, ED severity, and work status [20].

#### Secondary analyses

All patients prescribed tadalafil OaD at baseline were included in the longitudinal analyses. Least-square (LS) mean effects in IIEF domain scores and EDITS total score from baseline to Month 6 were assessed using a mixed model for repeated measures (MMRM), including the following prespecified variables as fixed effects: visit, age (18–65 vs >65 years), pretreatment with PDE5 inhibitors (yes vs no), etiology of ED, ED severity, BPH (yes vs no), diabetes (yes vs no), CVD (yes vs no), hypertension (yes vs no), and dyslipidemia (yes vs no). *p*-values <0.05 were considered statistically significant and 95 % CIs were produced. Data were analyzed using the SAS 9.2 software (SAS Institute Inc., Cary, NC, USA).

## Results

### Patient characteristics

Overall, 778 patients received prescriptions for initiating or switching to tadalafil OaD at baseline. The majority of patients were aged 18 to 65 years (76.9 %), had suffered from ED for at least 1 year (63.1 %), and presented with mild to moderate disease at baseline (73.7 %) (Table 1). Most patients (65.6 %) were naïve to PDE5 inhibitor treatment. Baseline IIEF domain scores were similar in PDE5 inhibitor-naïve and pretreated patients (Additional file 2). Over half of the patients (58.4 %) reported having at least 1 comorbid condition. The most commonly reported comorbidities included CVD (34.5 %), hypertension (33.4 %), dyslipidemia (18.5 %), and diabetes (15.9 %). A small proportion of patients reported BPH (6.3 %) and hypogonadism (1.5 %). Approximately 57.1 % of the patients were on at least 1 concomitant medication (Table 1).

**Table 1:** Baseline characteristics of patients starting tadalafil 5 mg OaD (*N* = 778)

Variable	N with data	
Age, years	778	
Median (IQR)		57 (47–65)
18 − 65 years, n (%)		598 (76.9)
Smoking habits, n (%)	775	
Current smoker		148 (19.0)
Former smoker		159 (20.4)
Never smoker		468 (60.2)
Currently drink alcohol, n (%)	775	456 (58.8)
ED severity, n (%)	775	
Mild		160 (20.6)
Moderate		411 (53.0)
Severe		204 (26.3)
Duration of ED symptoms, n (%)	776	
< 3 months		55 (7.1)
3 to <12 months		231 (29.7)
≥ 12 months		490 (63.1)
ED etiology, n (%)	776	
Mixed		343 (44.2)
Organic		240 (30.9)
Psychogenic		145 (18.7)
Unknown		48 (6.2)
With penile defects, n (%)	776	24 (3.1)
With former invasive diagnostic procedure for ED, n (%)	776	150 (19.3)
Non-coital erections, n (%)	771	422 (54.7)
Decreased libido, n (%)	775	310 (40.0)
IIEF-EF at baseline, mean (SD)	776	14.5 (7.06)
EDITS total score at baseline, mean (SD)	178^a^	59.6 (21.27)
Previous treatment, n (%)	777	
PDE5 inhibitor pretreated		267 (34.3)
PDE5 inhibitor-naïve		510 (65.6)
Relevant categories of comorbidities, n (%)	778	
At least 1 comorbidity		454 (58.4)
Cardiovascular disorder		268 (34.5)
Hypertension		260 (33.4)
Dyslipidemia		144 (18.5)
Diabetes		124 (15.9)
Pelvic surgery		89 (11.4)
Benign prostatic hyperplasia		49 (6.3)
Hypogonadism		12 (1.5)
Concomitant medication, n (%)	778	
At least 1 concomitant medication		444 (57.1)
Antihypertensive medication		260 (33.4)
Lipid lowering medication		152 (19.5)
Oral antidiabetic medication		102 (13.1)
Cardiovascular medication		94 (12.1)
α-blockers		58 (7.5)
5α-reductase inhibitors		14 (1.8)

*ED*, erectile dysfunction, *EDITS* Erectile Dysfunction Inventory of Treatment Satisfaction, *IIEF-EF* International Index of Erectile Function-Erectile Function, *IQR* inter quartile range, *N* number of patients with data, *n* number of patients, *OaD* once a day, *PDE5* phosphodiesterase type 5, *SD* standard deviation

^a^EDITS scores at baseline were collected only in patients pretreated with PDE5 inhibitors

### Impact of baseline characteristics and comorbidities on continuation rates of tadalafil OaD

At Month 2, Kaplan-Meier estimation revealed that more than 90 % of patients were still on tadalafil OaD, except those aged >65 years (86.7 %) and men with severe ED (89.0 %) (Table 2). More than 80 % of patients in all subgroups, except those aged >65 years (75.0 %) continued tadalafil OaD at Month 6 (Table 2; Additional file 3). Reasons for discontinuation did not differ notably between the older (>65 years) and younger age group (Table 3).

**Table 2:** Impact of patient characteristics and comorbidities on the continuation of treatment with tadalafil 5 mg OaD (*N* = 778)

Population	Number of patients with		Patients without events (%), KM estimate [95 % CI]		
	Data	Events	Month 2	Month 4	Month 6
Overall (primary analysis)	773	107	94.0 [92.3,95.7]	88.3 [85.9,90.6]	86.3 [83.7,88.9]
By age					
Age ≤65 years	593	61	**96.2 [94.7,97.8]**	**91.7 [89.4,93.9]**	**89.8 [87.1,92.4]**
Age >65 years	180	46	**86.7 [81.7,91.6]**	**77.3 [71.1,83.5]**	**75.0 [68.5, 81.5]**
By PDE5-I treatment					
PDE5-I naïve	507	71	93.9 [91.8,96.0]	88.5 [85.7,91.4]	86.1 [82.9,89.4]
PDE5-I pretreated	265	36	94.2 [91.4,97.1]	87.6 [83.6,91.7]	86.6 [82.3,90.9]
By disease severity					
Mild ED	160	16	**98.1 [96.0,1.00]**	**94.1 [90.3,97.8]**	89.8 [84.4,95.3]
Moderate ED	407	55	94.8 [92.7,97.0]	88.6 [85.5,91.8]	87.3 [83.9,90.8]
Severe ED	203	36	**89.0 [84.6,93.3]**	**82.8 [77.4,88.2]**	80.8 [75.1,86.5]
By presence of BPH					
BPH present	49	10	91.8 [84.2,99.5]	87.8 [78.6,96.9]	84.9 [74.5,95.3]
BPH absent	724	97	94.1 [92.4,95.9]	88.3 [85.9,90.7]	86.4 [83.7,89.1]
By presence of diabetes					
Diabetes present	123	19	93.4 [88.9,97.8]	85.3 [78.9,91.8]	84.0 [77.2,90.9]
Diabetes absent	650	88	94.1 [92.3,95.9]	88.8 [86.3,91.3]	86.7 [83.9, 89.5]
By presence of CVD					
CVD present	266	41	90.9 [87.4,94.4]	86.8 [82.7,91.0]	84.6 [80.1,89.2]
CVD absent	507	66	95.6 [93.8,97.4]	89.0 [86.2,91.8]	87.2 [84.0,90.3]
By presence of hypertension					
Hypertension present	258	40	91.0 [87.5,94.5]	86.8 [82.6,91.0]	84.6 [79.9,89.2]
Hypertension absent	515	67	95.5 [93.7,97.3]	89.0 [86.2,91.8]	87.2 [84.0,90.3]
By presence of dyslipidemia					
Dyslipidemia present	144	24	91.6 [87.0,96.1]	86.3 [80.6,92.1]	83.5 [77.2,89.9]
Dyslipidemia absent	629	83	94.5 [92.8,96.3]	88.7 [86.2,91.2]	87.0 [84.2,89.8]

Event = Discontinuation of tadalafil OaD

Note: KM estimates for subgroups with non-overlapping CIs at given time points are marked in bold

*BPH* benign prostatic hyperplasia, *CI* confidence interval, *CVD* cardiovascular diseases, *ED* erectile dysfunction, *KM* Kaplan-Meier, *N* number of patients, *OaD* once a day, *PDE5-I* phosphodiesterase type 5 inhibitor

**Table 3:** Reasons for discontinuation of tadalafil OaD treatment by age group

	Number (%) of patients		
	Age ≤65 years *N* = 598	Age >65 years *N* = 180	Overall *N* = 778
Discontinued tadalafil OaD^a^	61 (100.0)	46 (100.0)	107 (100.0)
Reasons			
Lack of efficacy (hardness of erection)	17 (27.9)	16 (34.4)	33 (30.8)
Adverse event	10 (22.5)	12 (26.1)	22 (20.6)
Cost of medication	11 (18.0)	5 (10.9)	16 (15.0)
Didn’t want to take a pill every day	7 (11.0)	5 (10.9)	12 (11.2)
Patient discontinued study	7 (11.5)	2 (4.3)	9 (8.4)
Partner’s request	2 (3.3)	3 (6.5)	5 (4.7)
Felt that medication controlled his sexual life	2 (4.2)	1 (2.2)	3 (2.8)
Slow onset of action	2 (3.3)	1 (2.2)	3 (2.8)
Lack of efficacy (duration of erection)	1 (1.6)	1 (2.2)	2 (1.9)
Lack of confidence in medication	1 (1.6)	0	1 (0.9)
Non-desired spontaneous erections	1 (1.6)	0	1 (0.9)

*N* number of patients, *OaD* once a day

^a^Includes all patients with documented end date of tadalafil OaD treatment, irrespective if the patient completed or discontinued the study

As the majority of patients remained on tadalafil OaD at Month 6, the median time to discontinuation could not be estimated in any of the subgroups. Patients with severe ED had lower treatment continuation rates than those with mild ED or moderate ED, but these were still >80 % at all time points (Additional file 4). The Kaplan-Meier analysis did not suggest any influence of individual comorbidities on treatment continuation rates (Table 2).

These findings were consistent with those from the Cox proportional hazard model evaluating factors associated with time to discontinuation of tadalafil OaD. The risk of discontinuation was significantly decreased among younger vs older patients (HR [95 % CI]: 18–65 vs >65 years: 0.54 [0.30, 0.96], *p* = 0.038). There was no significant effect of disease severity (mild vs moderate ED: 1.26 [0.68, 2.32], *p* = 0.462; severe vs moderate ED: 1.51 [0.95, 2.39], *p* = 0.077), or the presence of relevant comorbidities (presence vs absence of relevant comorbidities, namely, BPH, diabetes, CVD, hypertension, dyslipidemia: 1.27 [0.82, 1.94], *p* = 0.287) in this model.

### Impact of patient characteristics and comorbidities on erectile function and treatment satisfaction during treatment with tadalafil OaD

Overall, treatment with tadalafil OaD for 6 months was associated with significant improvement at Visit 2 (1–3 months) and Visit 3 (4–6 months) in all IIEF domain scores, including the IIEF-erectile function (EF) domain (*p* < 0.001 for both visits), as well as the EDITS total score (*p* = 0.035 and *p* = 0.028 for Visits 2 and 3, respectively) (Table 4). Improvements in the mean (standard deviation [SD]) IIEF domain scores and EDITS total score are given in Additional file 5.

**Table 4:** Impact of patient-characteristics and comorbidities on EF and treatment-satisfaction during tadalafil OaD treatment (*N* = 778; MMRM)

	IIEF domain scores (*n* = 646)					EDITS total score (*n* = 165)^a^
	Erectile function	Orgasmic function	Sexual desire	Intercourse satisfaction	Overall satisfaction	
Change from baseline at Visit 2						
LS mean	+6.17*	+1.06*	+0.38*	+2.70*	+2.26*	+14.17*
95 % CI	+4.82 to +7.51	+0.54 to +1.57	+0.03 to +0.74	+2.02 to +3.38	+1.77 to +2.75	+1.00 to +27.34
*p*-value	<0.001	<0.001	0.034	<0.001	<0.001	0.035
Change from baseline at Visit 3						
LS mean	+7.11*	+1.36*	+0.48*	+3.18*	+2.56*	+14.83*
95 % CI	+5.76 to +8.47	+0.83 to +1.88	+0.12 to +0.84	+2.48 to +3.87	+2.06 to +3.06	+1.64 to +28.02
*p*-value	<0.001	<0.001	0.010	<0.001	<0.001	0.028
Fixed effects						
Age group (18–65 vs >65 years)						
LS mean effect	+0.45	−0.11	+0.15	+0.20	+0.04	+11.25*
95 % CI	−0.98 to +1.88	−0.66 to +0.44	−0.23 to +0.53	−0.53 to +0.92	−0.49 to +0.56	+2.96 to +19.54
*p*-value	0.539	0.707	0.444	0.592	0.896	0.008
PDE5-I pretreatment (yes vs no)						
LS mean effect	−2.13*	−0.69*	−0.10	−0.93*	−0.74*	+7.11
95 % CI	−3.33 to −0.94	−1.15 to −0.23	−0.41 to +0.22	−1.54 to −0.33	−1.17 to −0.30	−12.5 to +26.74
*p*-value	<0.001	0.003	0.548	0.003	0.001	0.475
ED etiology						
Psychogenic vs mixed						
LS mean effect	−0.25	+0.17	+0.04	−0.03	+0.06	−4.69
95 % CI	−1.75 to +1.26	−0.41 to +0.75	−0.35 to +0.44	−0.79 to +0.74	−0.49 to +0.62	−15.2 to +5.79
*p*-value	0.747	0.558	0.825	0.944	0.817	0.378
Organic vs mixed						
LS mean effect	−0.63	−0.17	−0.13	−0.44	−0.39	−1.70
95 % CI	−1.97 to +0.71	−0.69 to +0.34	−0.48 to +0.23	−1.12 to +0.24	−0.88 to +0.10	−9.31 to +5.92
*p*-value	0.359	0.507	0.479	0.205	0.116	0.661
ED severity (investigator assessment)						
Mild vs moderate						
LS mean effect	−2.00*	−0.06	−0.45*	−0.56	−0.97*	−4.52
95 % CI	−3.54 to −0.46	−0.66 to +0.53	−0.86 to −0.05	−1.35 to +0.22	−1.53 to −0.41	−16.4 to +7.35
*p*-value	0.011	0.833	0.029	0.156	<0.001	0.453
Severe vs moderate						
LS mean effect	+0.99	+0.93*	−0.21	+0.30	+0.30	+3.99
95 % CI	−0.37 to +2.34	+0.41 to +1.45	−0.57 to +0.15	−0.39 to +0.99	−0.20 to +0.80	−3.37 to +11.35
*p*-value	0.154	<0.001	0.260	0.395	0.237	0.285
Comorbidities						
BPH (yes vs no)						
LS mean effect	−2.77*	−0.87*	−0.72*	−1.08	−0.52	+5.78
95 % CI	−4.98 to −0.55	−1.73 to −0.02	−1.30 to −0.13	−2.21 to +0.04	−1.34 to +0.29	−8.38 to +19.94
*p*-value	0.014	0.044	0.017	0.058	0.206	0.421
Diabetes (yes vs no)						
LS mean effect	+2.28*	+0.81*	+0.06	+0.92*	+0.81*	+5.62
95 % CI	+0.64 to +3.92	+0.17 to +1.44	−0.38 to +0.50	+0.09 to +1.76	+0.20 to +1.41	−3.62 to +14.71
*p*-value	0.007	0.013	0.787	0.030	0.009	0.223
CVD (yes vs no)						
LS mean effect	−1.78	−1.93	0	−0.97	−0.49	+3.48
95 % CI	−7.53 to +3.96	−4.14 to +0.29	−1.52 to +1.52	−3.88 to +1.95	−2.60 to +1.62	−2.70 to +33.97
*p*-value	0.542	0.088	0.997	0.515	0.648	0.822
Hypertension (yes vs no)						
LS mean effect	+1.62	+1.91	+0.29	+0.77	−0.04	−1.33
95 % CI	−4.14 to +7.39	−0.31 to +4.13	−1.24 to +1.81	−2.16 to +3.69	−2.15 to +2.07	−31.7 to +29.00
*p*-value	0.580	0.091	0.711	0.606	0.969	0.931
Dyslipidemia (yes vs no)						
LS mean effect	−0.05	−0.19	−0.31	+0.02	−0.17	−4.26
95 % CI	−1.61 to +1.51	−0.79 to +0.41	−0.72 to +0.11	−0.78 to +0.81	−0.74 to +0.41	−13.1 to +4.60
*p*-value	0.946	0.528	0.145	0.966	0.568	0.343

*BPH* benign prostatic hyperplasia, *CI* confidence interval, *CVD* cardiovascular disease, *ED* erectile dysfunction, *EDITS* Erectile Dysfunction Inventory of Treatment Satisfaction, *IIEF* International Index of Erectile Function, *LS mean* least-square mean, *MMRM* Mixed Model for Repeated Measures, *N* number of patients, *n* number of patients included in the model, *OaD* once a day, *PDE5-I* phosphodiesterase type 5 inhibitor

^**a**^EDITS scores at baseline were collected only in patients pretreated with PDE5-I

*95 % confidence interval does not include zero and *p*-value statistically significant (<0.05)

MMRM analysis showed that PDE5 pretreatment had a significantly smaller LS mean effect on IIEF-EF (*p* < 0.001), orgasmic function (*p* = 0.003), intercourse satisfaction (*p* = 0.003), and overall satisfaction (*p* = 0.001) domain scores vs no previous PDE5 inhibitor treatment. Mild ED had a significantly smaller LS mean effect on IIEF-EF (*p* = 0.011), sexual desire (*p* = 0.029), and overall satisfaction (*p* < 0.001) domain scores, while severe ED had a significantly increased LS mean effect on the IIEF orgasmic function (*p* < 0.001) domain score relative to moderate ED. The negative effect of BPH on change in IIEF-EF (*p* = 0.014), orgasmic function (*p* = 0.044), and sexual desire (*p* = 0.017) domain scores was statistically significant. Furthermore, diabetes showed a significant positive effect on changes in IIEF-EF (*p* = 0.007), orgasmic function (*p* = 0.013), intercourse satisfaction (*p* = 0.030), and overall satisfaction (*p* = 0.009) domain scores (Table 4).

There were no significant effects for age, ED etiology, CVD, hypertension, or dyslipidemia (*p* > 0.05 for all comparisons) on IIEF domain scores over 6 months of tadalafil OaD treatment. LS mean effects in EDITS total score were not affected by any comorbidity or baseline characteristic except age (*p* = 0.008) (Table 4).

### Safety

No treatment-related serious adverse events were reported during the study. The most frequently reported adverse events included headache (1.3 %), dyspepsia (0.5 %), and a new diagnosis of BPH (0.5 %); no new or unexpected safety signals were observed [20].

## Discussion

Currently, there are few observational studies in patients with ED that assess the impact of baseline characteristics and presence of comorbid conditions on PDE5 inhibitor treatment continuation rates. In particular, few existing studies assess treatment satisfaction and effectiveness beyond the IIEF-EF domain. The primary analysis of the EDATE observational study demonstrated high treatment continuation rates (86.3 %) over a period of 6 months in 778 patients with ED who initiated or switched to PDE5 inhibitor treatment with tadalafil OaD at baseline (Additional file 6) [20].

Here in this cohort with high frequency of comorbidities and use of concomitant medications, we found that continuation rates over 6 months of treatment with tadalafil OaD were comparable across subgroups. Furthermore, continuation rates were over 80 % regardless of the presence of comorbidities, pretreatment with PDE5 inhibitors, or ED severity. Even in the subgroup of older patients (>65 years), the continuation rate was 75 % at Month 6. IIEF was affected by some baseline characteristics including ED severity, pretreatment with PDE5 inhibitors, and the presence or absence of diabetes and BPH. However, treatment satisfaction remained predominantly unaffected by any comorbid condition or baseline characteristic except for age, with patients aged 18–65 years reporting significantly higher satisfaction scores vs those aged >65 years.

In our study, younger patients (18–65 years) had higher chances of continuing tadalafil OaD vs older patients. This finding resonates with that from the DETECT observational study in which age less than 60 years was one of the factors associated with continuation of tadalafil on-demand treatment at 12 months [23]. Similarly, an observational study in Latin America found that persistence and adherence rates for PDE5 inhibitor treatment at 6 months were generally higher in younger men with ED (mean age, 52.3 years vs 54.9 years for non-persistent patients and 52.1 years vs 55.5 years for non-adherent patients) [24]. In a Korean study, the most common reason for discontinuing PDE5 inhibitor treatment given by older men (>70 years) with ED was concerns about the side effects, possibly due to higher prevalence of comorbidities in this subgroup [25].

We observed significant improvement from baseline in all the IIEF domain scores and EDITS total score at the second (1–3 months) and third (4–6 months) visits in the overall tadalafil OaD cohort. In particular, the LS mean IIEF-EF domain score increased by 7.1 points from baseline to 4 to 6 months after initiation of tadalafil OaD, exceeding the minimal clinically important difference (MCID) of 4 points [26]; this observation aligns with the 9.4-point increase observed with tadalafil OaD in a previous randomised controlled trial [27].

We found that PDE5 inhibitor pretreatment had a significant impact on IIEF scores, i.e. pretreated patients showed less improvement during tadalafil OaD treatment compared with treatment-naïve patients, in the domains of EF, orgasmic function, intercourse satisfaction, and overall satisfaction. In the EDATE cohort, 36.7 % of patients switched to tadalafil OaD due to lack of efficacy of previous PDE5 inhibitor treatment [20]. Thus, we believe that some of the patients in the tadalafil OaD cohort who were pretreated with PDE5 inhibitors may have been insufficient responders to any PDE5 inhibitor treatment, leading to a negative effect on their IIEF domain scores. In real-life clinical practice, men with inadequate response to ED treatment will usually keep seeking for a better treatment option to improve their erection. A large observational study in Middle Eastern countries among men with ED receiving tadalafil on-demand (*N* = 1,080) also found significantly higher mean IIEF-EF domain scores in treatment-naïve patients vs those who were pretreated (13.26 vs 9.28; *p* < 0.0001) [28].

ED etiology had no significant effect on IIEF in our study. This is in keeping with findings from an integrated analysis of 6 randomised studies which showed that tadalafil OaD was consistently efficacious across disease etiologies in men with ED [29].

In this study, there were significant effects of ED severity on the IIEF-EF, orgasmic function, sexual desire, and overall satisfaction domains. Mild ED reduced the change from baseline for IIEF-EF, sexual desire, and overall satisfaction, while severe ED meant an increased change in orgasmic function relative to moderate ED. This aligns with findings from an analysis of 6 randomised studies of tadalafil 2.5 and 5 mg OaD in patients with ED. Placebo-adjusted LS mean changes in IIEF-EF from baseline for both doses of tadalafil were numerically higher in patients with severe ED (4.6 and 5.8) vs those in patients with mild (2.3 and 4.5) and moderate (4.4 and 5.5) ED [29]. This may be explained by the possibility of greater changes from baseline in patients with more severe disease compared with those with mild or moderate disease. Furthermore, Rosen and colleagues have demonstrated that the MCID in the IIEF-EF domain increases significantly with higher baseline severity of ED (*p* < 0.0001) [26].

We observed a significant impact of reported BPH on IIEF-EF, orgasmic function, and sexual desire domain scores, i.e., the presence of BPH reduced improvement during tadalafil OaD treatment. However, BPH can be accompanied by other comorbidities and the estimated reduction in efficacy from our model is unlikely to define the differences between BPH and non-BPH patients alone. It is worth noting that patients with BPH in our study did show improvement from baseline to Month 6 in unadjusted mean (SD) IIEF-EF domain score (5.7 [6.7]); Additional file 5). However, self-reported prevalence of BPH was small (6.3 %), and a full baseline assessment of LUTS/BPH was not actively performed. Thus, our findings in the small number of patients with BPH should be interpreted with caution.

In our study, diabetes significantly impacted the changes in IIEF domains of EF, orgasmic function, intercourse satisfaction, and overall satisfaction, i.e., patients with diabetes showed more improvement than patients without diabetes. This may have occurred because patients with diabetes had more severe ED at baseline. According to the MALES study, men with diabetes were more likely to perceive their ED to be severe and permanent vs those without diabetes [30]. The efficacy of tadalafil OaD for ED treatment in males with diabetes has been previously evaluated in a placebo controlled, randomised multicentre study, showing statistically significant improvements in the IIEF-EF (*p* ≤ 0.005), intercourse satisfaction (*p* = 0.033), and overall satisfaction (*p* < 0.001) domain scores vs placebo [31].

There was no significant effect of CVD, dyslipidemia, and hypertension on any of the IIEF domain scores, similar to results from previous studies. In an integrated analysis of data from 6 randomised controlled trials, patients with ED receiving tadalafil 2.5 and 5 mg OaD experienced IIEF-EF LS mean improvements reaching or exceeding the MCID (≥4) regardless of CVD, diabetes, hypertension, or hyperlipidemia [29]. Furthermore, in an analysis of 11 placebo-controlled randomised studies, tadalafil on-demand significantly improved IIEF-EF domain score from baseline (*p* < 0.005 vs placebo) in all subpopulations regardless of age, disease etiology, severity and duration, and presence of comorbid conditions such as diabetes, hypertension, CVD, hyperlipidemia, depression, and BPH [32].

Treatment satisfaction, as assessed by EDITS, was not affected by pretreatment with PDE5 inhibitors, ED etiology, ED severity, or presence of relevant comorbidities in this study. This is consistent with data from a previous randomised study in which patients receiving fixed-dose tadalafil on-demand were significantly more satisfied with their treatment vs placebo (*p* < 0.001) despite high incidence of severe, organic ED and presence of comorbid conditions [33].

Large overall sample size, longitudinal nature, real life setting, and including patients with ED from several countries were important strengths of this study. In addition, to increase the external validity of the study, study sites were selected randomly from a list of investigators who expressed interest in participation and by asking sites to enroll patients consecutively. However, results may still be biased as only sites and investigators with interest were selected. As patients had to pay for their treatment, the study may also be biased towards those with higher education and economic status. The prevalence of comorbidities was based on patient-reported data, and was not confirmed by active baseline assessment by investigators. Thus, results may have been biased by false positive or missed diagnoses, and should be interpreted with caution.

Being an observational study, the patient population within subgroups was not well-defined. The small number of discontinuation events additionally limits the subgroup analysis of time to discontinuation. Nevertheless, this study provides valuable data on the impact of baseline characteristics and presence of comorbidities on continuation rates, and effectiveness and satisfaction of treatment with tadalafil OaD in a naturalistic setting and therefore has implications for everyday clinical practice. Furthermore, ED and CVD have common pathogenic pathways mediated via endothelial dysfunction [34], and it is suggested that tadalafil may potentially provide beneficial effects in patients with coronary artery disease, hypertension, heart failure, pulmonary arterial hypertension, diabetes mellitus, and Raynaud’s phenomenon [35].

## Conclusions

This observational study found that under routine clinical conditions, the presence of comorbidities did not have any significant impact on treatment continuation rates and treatment satisfaction among men with ED who choose treatment with tadalafil OaD. Continuation rates and treatment satisfaction were comparatively lower in men older than 65 years. Pretreatment with PDE5 inhibitors, ED severity, and presence or absence of diabetes and BPH had an impact on treatment effectiveness; however, age and presence of hypertension, CVD, dyslipidemia, or ED etiology did not affect IIEF in this cohort. These findings have clinical implications for counseling patients with ED, particularly for discussing expectations after PDE5 inhibitor treatment, as comorbidities are common in these patients, especially in older men.

## Additional files

Additional file 1:
**List of ethical review boards.** (PDF 17 kb) Additional file 2:
**IIEF domain scores at baseline in PDE5 inhibitor-naïve and PDE5 inhibitor pretreated patients (** ***N*** **= 778).** (PDF 24 kb) Additional file 3:
**Kaplan-Meier estimation in different age groups for time to discontinuation of tadalafil OaD treatment.** (PDF 109 kb) Additional file 4:
**Kaplan-Meier estimation in patients stratified according to severity of erectile dysfunction for time to discontinuation of tadalafil OaD treatment.** (PDF 118 kb) Additional file 5:
**Change of IIEF and EDITS total scores from baseline to Month 6 during treatment with tadalafil OaD - subgroup analysis (** ***N*** **= 778; unadjusted data).** (PDF 74 kb) Additional file 6:
**Kaplan-Meier estimation for time to discontinuation of tadalafil OaD treatment.** (PDF 102 kb)

## Abbreviations

BPH: benign prostate hyperplasia
CI: confidence interval
CVD: cardiovascular disease
ED: erectile dysfunction
EDITS: Erectile Dysfunction Inventory of Treatment Satisfaction
EF: erectile function
HR: hazard ratio
IIEF: International Index of Erectile Function
LS mean: least squares mean
LUTS: lower urinary tract symptoms
MCID: minimal clinically important difference
MMRM: mixed model for repeated measures
OaD: once daily
PDE5-inhibitor: phosphodiesterase-type-5 inhibitor
SD: standard deviation
