# Supplementary material for: Tadalafil 5 mg once daily for the treatment of erectile dysfunction during a 6-month observational study (EDATE): impact of patient characteristics and comorbidities
Source: BMC Urol. 2015 Nov 12;15:111. doi: 10.1186/s12894-015-0107-5 (PMC4643510; doi:10.1186/s12894-015-0107-5)
Supplement: Additional file 1: — List of ethical review boards. (PDF 17 kb) [file 12894_2015_107_MOESM1_ESM.pdf]

Additional file 1. Listing of Ethical Review Boards.

Name of Ethic Comittee	Address	Postcode	City, Country
Ethikkommission der Ärztekammer Hamburg	Weidestr. 122 b	22083	Hamburg, Germany
Comitato Etico della Provincia di Bergamo	Piazza OMS- Organizzazione Mondiale della Sanita 1	24128	Bergamo, Italy
Comitato Etico Azienda Ospedaliero-Universitaria di Bologna	Via Albertoni, 15	40138	Bologna, Italy
Comitato Etico Ospedaliero-Universitaria di Bologna-Imola	Largo B. Nigrisoli, 2	40133	Bologna, Italy
Comitato Etico dell` Azienda Ospedaliero-Universitaria Riuniti di Foggia	Viale Luigi Pinto	71100	Foggia, Italy
Comitato Etico Ospdaliero S. Paolo di Milano	Via Di Rudini, 8	20142	Milano, Italy
Comitato Etico della Provincia di Modena	Via Largo del Pozzo, 71	41124	Modena, Italy
Comitato Etico Provinciale c/o Azienda Policlinico	Via del Pozzo 71	41100	Modena, Italy
Comitato Etico Azienda Ospedaliera San Gerardo	Via Pergolesi, 33	20052	Monza, Italy
Comitato Etico Area Vasta Nord Ovest presso Azienda Ospedaliero-Universitaria Pisana	Via Roma, 67	56126	Pisa, Italy
Comitato Etico Indipendente ricostituito presso la Fondazione PTV Policlinico Tor Vergata	Viale Oxford, 81	00133	Rome, Italy
Comitato Etico Lazio l presso Azienda Ospedaliero S.Camillo-Forlanini	Circonvallazione Gianicolense, 87	00152	Rome, Italy
Comitato Etico del Policlinico Gemelli	Largo Agostino Gemelli, 8	00168	Rome, Italy
Comitato Etico dell'Azienda Ospedaliero-Universitaria di Sassari	Via Michele Coppino, 26	07100	Sassari, Italy
Comitato Etico Aziendale dell'ASL 2 Savonese di Savona	Via Manzoni, 14	17100	Savona, Italy
Comitato Etico Area Vasta Sud Est presso Azienda Ospedaliero-Universitaria Senese, c/o U.O.C. Farmacia AOUS	Viale Bracci, 16	53100	Siena, Italy
Comitato Bioetico Aziendale della AUSL 8 di Siracusa	Corso Gelone, 17	96100	Siracusa, Italy
Comitato Etico Indipendente dell'Azienda Ospedaliero-Universitaria Degli Ospedali Riuniti di Trieste	Via del Farneto 3	34142	Trieste, Italy
Comitato Etico per la Sperimentazione Clinica della Provincia di Verona	Via Salvo d'Acquisto, 7	37122	Verona, Italy
