# Supplementary material for: Tadalafil 5 mg once daily for the treatment of erectile dysfunction during a 6-month observational study (EDATE): impact of patient characteristics and comorbidities
Source: BMC Urol. 2015 Nov 12;15:111. doi: 10.1186/s12894-015-0107-5 (PMC4643510; doi:10.1186/s12894-015-0107-5)
Supplement: Additional file 2: — IIEF domain scores at baseline in PDE5 inhibitor-naïve and PDE5 inhibitor pretreated patients ( N = 778). (PDF 24 kb) [file 12894_2015_107_MOESM2_ESM.pdf]

Additional file 2. IIEF domain scores at baseline in PDE5 inhibitor-naïve and PDE5 inhibitor pretreated patients (N=778)

Mean (SD) score at baseline	PDE5 inhibitor naïve N=510[a]	PDE5 inhibitor pretreated N=267[a]
**IIEF domain scores**		
Erectile function	14.3 (6.99)	14.7 (7.20)
Orgasmic function	6.5 (3.22)	6.6 (3.10)
Sexual desire	6.6 (1.90)	6.7 (1.88)
Intercourse satisfaction	6.7 (3.62)	6.9 (3.87)
Overall satisfaction	4.7 (2.29)	5.0 (2.48)

[a] For 1 patient, PDE5 inhibitor pretreatment status was unknown.
IIEF, International Index of Erectile Function; PDE5, phosphodiesterase type 5; SD, standard deviation
