# Supplementary material for: Tadalafil 5 mg once daily for the treatment of erectile dysfunction during a 6-month observational study (EDATE): impact of patient characteristics and comorbidities
Source: BMC Urol. 2015 Nov 12;15:111. doi: 10.1186/s12894-015-0107-5 (PMC4643510; doi:10.1186/s12894-015-0107-5)
Supplement: Additional file 5: — Change of IIEF and EDITS total scores from baseline to Month 6 during treatment with tadalafil OaD - subgroup analysis ( N = 778; unadjusted data). (PDF 74 kb) [file 12894_2015_107_MOESM5_ESM.pdf]

Additional file 5. Change of IIEF and EDITS total scores from baseline to Month 6 during treatment with tadalafil OaD - subgroup analysis (N=778; unadjusted data)

Variable	Mean (SD) score change from baseline to Month 6					
	IIEF domain scores					EDITS total score[a] n=183
	Erectile function n=776	Intercourse satisfaction n=776	Orgasmic function n=776	Overall satisfaction n=776	Sexual desire n=776	
**By age**						
Age ≤65 years	8.6 (7.9)	3.7 (4.1)	1.6 (2.9)	2.9 (2.9)	1.0 (2.0)	17.9 (23.3)
Age >65 years	8.0 (7.3)	3.3 (3.8)	1.6 (3.4)	2.8 (2.9)	0.8 (2.2)	11.5 (17.5)
**By PDE5-I treatment**						
PDE5-I naïve	9.1 (7.7)	3.9 (3.9)	1.8 (3.0)	3.1 (2.8)	1.0 (2.0)	4.5 (17.7)
PDE5-I pretreated	7.4 (7.9)	3.1 (4.2)	1.3 (3.0)	2.5 (3.0)	0.9 (2.1)	16.6 (22.1)
**By disease severity**						
Mild ED	6.2 (6.9)	2.8 (4.1)	1.2 (2.9)	2.2 (2.8)	0.6 (1.9)	10.9 (19.5)
Moderate ED	8.6 (7.6)	3.7 (3.6)	1.5 (2.9)	2.9 (2.8)	1.1 (2.0)	15.2 (21.1)
Severe ED	10.2 (8.6)	4.2 (4.8)	2.5 (3.3)	3.5 (3.0)	1.0 (2.2)	20.3 (24.4)
**By presence of BPH**						
BPH present	5.7 (6.7)	2.5 (3.2)	0.8 (3.1)	2.5 (2.8)	0.0 (2.2)	14.2 (18.5)
BPH absent	8.7 (7.9)	3.7 (4.1)	1.7 (3.0)	2.9 (2.9)	1.0 (2.0)	16.5 (22.4)
**By presence of diabetes**						
Diabetes present	9.8 (7.6)	4.0 (4.2)	2.2 (3.4)	3.1 (2.9)	0.9 (1.9)	21.6 (22.9)
Diabetes absent	8.2 (7.8)	3.6 (4.0)	1.5 (3.0)	2.8 (2.9)	0.9 (2.1)	15.1 (21.8)
**By presence of CVD**						
CVD present	8.2 (8.0)	3.3 (4.0)	1.6 (3.4)	2.6 (3.0)	1.0 (2.1)	17.8 (24.3)
CVD absent	8.6 (7.7)	3.8 (4.1)	1.7 (2.8)	3.0 (2.8)	0.9 (2.0)	15.3 (20.6)
**By presence of hypertension**						
Hypertension present	8.3 (8.0)	3.3 (4.0)	1.7 (3.4)	2.6 (3.0)	1.0 (2.1)	18.3 (24.2)
Hypertension absent	8.6 (7.7)	3.8 (4.0)	1.6 (2.8)	3.0 (2.8)	0.9 (2.0)	15.0 (20.6)
**By presence of dyslipidemia**						
Dyslipidemia present	8.3 (8.3)	3.4 (4.3)	1.5 (3.3)	2.7 (2.9)	0.8 (2.1)	13.6 (25.3)
Dyslipidemia absent	8.5 (7.7)	3.7 (4.0)	1.7 (3.0)	2.9 (2.9)	1.0 (2.0)	17.0 (21.2)

[a] EDITS scores at baseline were collected only in patients pretreated with PDE5-I

BPH, benign prostatic hyperplasia; CVD, cardiovascular disease; ED, Erectile Dysfunction; EDITS, Erectile Dysfunction Inventory of Treatment Satisfaction; IIEF, International Index of Erectile Function; N, number of patients; n, number of patients with data at baseline; OaD, once a day; PDE5-I, phosphodiesterase type 5 inhibitor; SD, standard deviation
