# Supplementary material for: Tadalafil 5 mg once daily for the treatment of erectile dysfunction during a 6-month observational study (EDATE): impact of patient characteristics and comorbidities
Source: BMC Urol. 2015 Nov 12;15:111. doi: 10.1186/s12894-015-0107-5 (PMC4643510; doi:10.1186/s12894-015-0107-5)
Supplement: Additional file 6: — Kaplan-Meier estimation for time to discontinuation of tadalafil OaD treatment. (PDF 102 kb) [file 12894_2015_107_MOESM6_ESM.pdf]

Additional file 6. Kaplan-Meier estimation of time to discontinuation of tadalafil OaD treatment

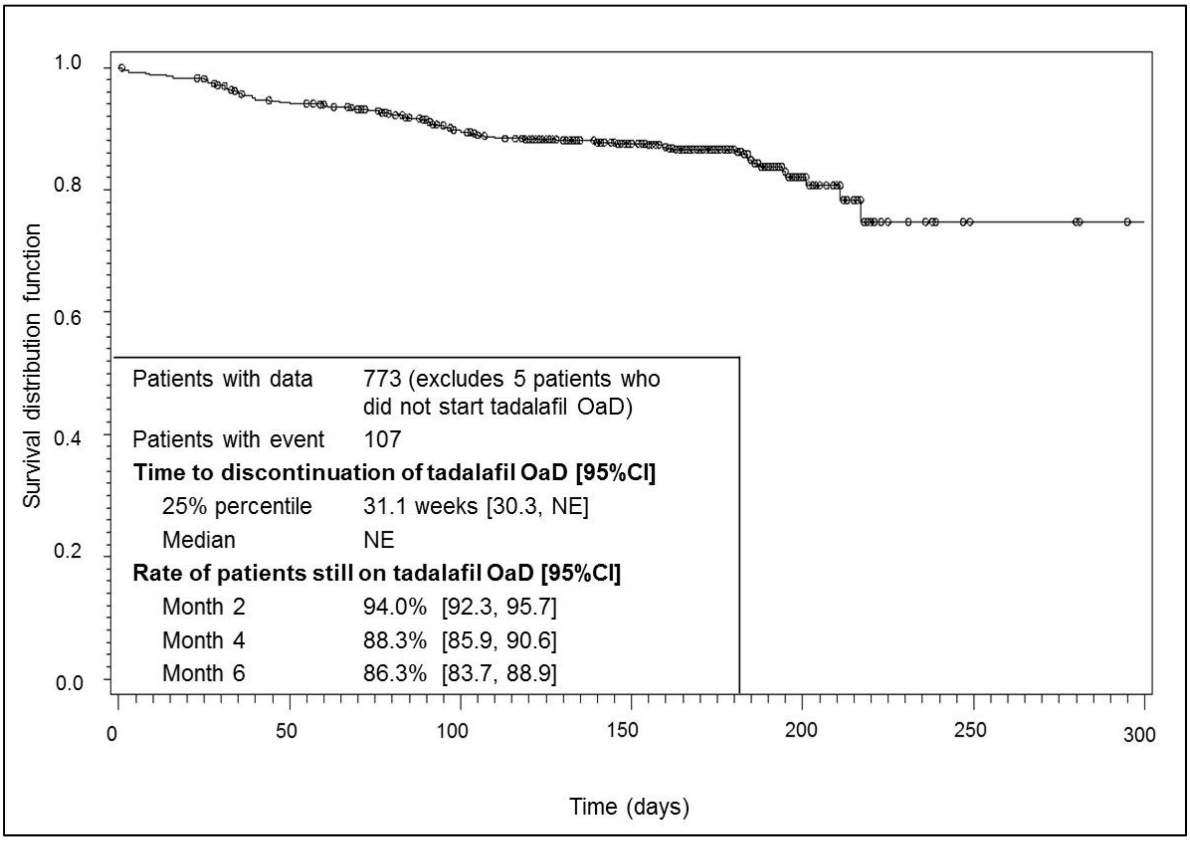

CI, confidence interval; NE, not estimable; OaD, once daily
